# Charles Bonnet Syndrome Following a Mild Traumatic Brain Injury

**DOI:** 10.7759/cureus.70638

**Published:** 2024-10-01

**Authors:** Christina Campbell, Ranita H Manocha, Vivian Hill, Chantel T Debert

**Affiliations:** 1 Department of Clinical Neurosciences, Cumming School of Medicine, University of Calgary, Calgary, CAN; 2 Department of Surgery, Cumming School of Medicine, University of Calgary, Calgary, CAN

**Keywords:** charles bonnet syndrome, concussion, mild traumatic brain injury, ophthalmology, post-concussion symptoms

## Abstract

Charles Bonnet syndrome (CBS) describes the presence of hallucinations in patients with poor or deteriorating vision. The physician awareness of CBS is low, despite reporting of CBS hallucinations occurring in a range of ocular and central nervous system conditions. Following mild traumatic brain injury (mTBI), patients can experience visual or oculomotor dysfunction. As such, it is possible that CBS might present following mTBI.

An adult male suffered an mTBI and whiplash injury following a motor vehicle accident. He developed persistent post-concussion symptoms (PPCS) including headaches and vestibular and visual disturbances. He reported experiencing visual hallucinations eight months post-mTBI and was diagnosed with CBS. Interventions for PPCS and hallucinations have persisted for five years. The treatment options for CBS are limited and the patient’s PPCS made participating in visual therapy challenging. Physicians assessing patients following mTBI should be aware of CBS and should take PPCS into consideration when recommending treatments for CBS.

## Introduction

Charles Bonnet syndrome (CBS) is characterized by the presence of temporary complex visual hallucinations in a person who has experienced a partial or complete loss of vision in the absence of an underlying psychiatric or behavioral disorder [1]. In Canada, as many as one in five patients with low or deteriorating vision may experience CBS [2]. However, the true incidence is likely higher as many patients may be reluctant to report hallucinations for fear they are not taken seriously or stigmatized with a label of mental illness [2].

Although CBS is more commonly reported in elderly patients with age-related macular degeneration, it has also been reported across different age ranges [3] and associated with a number of conditions such as stroke [4] and optic neuritis [5]. In addition, CBS has been reported in cases with relatively low vision loss [2] or where the visual field is impaired, but acuity is intact [6]. This may suggest that CBS is not a result of a specific condition or aging [2]. Although the exact pathophysiology of CBS remains unknown, there are two proposed theories: the release theory and the deprivation theory. The release theory postulates that impairment of the visual system interferes with normal circuitry within the visual cortex and visual hallucinations are “released” due to reduced inhibitory activity [7,8]. Conversely, the deprivation theory proposes that reduced visual input results in spontaneous compensatory excitability in the visual cortex, causing hallucinations [7,8].

Following mild traumatic brain injury (mTBI), approximately 30% of adults develop persistent post-concussion symptoms (PPCS) [9]. Of these patients, visual disturbances such as convergence insufficiency, accommodation disorders, and saccadic dysfunction are reported in up to 69% of participants in some studies [10,11]. The presence of these symptoms may reduce visual input or acuity; therefore, it is possible that CBS may occur following mTBI. Two cases of CBS following TBI have been reported in the literature. The first, a 66-year-old male, experienced visual hallucinations following a TBI due to a motor vehicle collision and was later diagnosed with Alzheimer’s [12]. The second case involved a 43-year-old male with bilateral eye blindness secondary to retinitis pigmentosa who was diagnosed with CBS whilst hospitalized for acute disorganized behavior, aggression, and hallucinations approximately one-month post-TBI [13]. Neither case clearly specified the severity of TBI.

Despite the increased risk of CBS with visual impairment, physician awareness of the condition has been shown to be as low as 45% [14]. This is problematic, as patients with no prior knowledge of CBS at hallucination onset or patients provided with limited information from healthcare professionals have demonstrated worse outcomes [15]. By presenting a patient diagnosed with CBS and PPCS following mTBI with no underlying psychiatric or behavioral disorders, we aim to increase awareness of CBS amongst physicians treating patients following concussion.

## Case presentation

A male in his 40s was stationary in his car at a traffic light when he was rear-ended by another vehicle. He does not remember losing consciousness and maintained a Glasgow Coma Scale of 15 but was amnestic for a few seconds with transient neurological dysfunction. Within 24 hours, he experienced headaches, back pain, and tinnitus. After returning to work the day after the accident, he was unable to concentrate on routine tasks and felt confused. His headaches progressively worsened over the next seven days, resulting in time off work. Approximately one week later, he was diagnosed with an mTBI and whiplash injury by his family doctor using the American Congress of Rehabilitation criteria [16]. His past medical history included hypothyroidism and prior to the mTBI, the patient was taking levothyroxine (150mg daily). There was no family history of neurological or ocular disease. The patient was a non-smoker, did not drink alcohol, and did not use drugs. He had completed post-secondary education.

In the weeks following the accident, he continued to have persistent symptoms including dizziness, fatigue, blurred vision, headaches, nasal discharge, tinnitus, irritability, photophobia, and phonophobia. As his symptoms persisted for over a month post-injury, he met the criteria for PPCS using the International Statistical Classification of Diseases 10th revision (ICD-10) criteria. Later that month, during an annual eye exam, the patient also reported experiencing right eye floaters and reported blurry vision with flashes of light in his periphery. His visual acuity with correction was 20/15-1 and 20/15-2. His glasses prescription was -6.50/+1.50 x 93 and -7.50/ +2.00 x 74. During a follow-up appointment for convergence insufficiency four months post-injury, he presented with a small limitation of abduction in the right eye and minimal esotropia in addition to lid fluttering and upgaze nystagmus. The ophthalmologist hypothesized that symptoms may be a result of a cranial nerve VI palsy from the mTBI. His neurological motor and sensory examinations were normal.

Eight months post-injury, the patient reported to ophthalmology that he had been missing things, for example, looking for a person or an object that was in front of him, problems with reading (missing words and lines), and halo/glare when looking at objects for a long time. During a visual field assessment, whilst looking at a dot on a plain background, the patient reported seeing a child in a pink dress having their diaper changed followed by a hockey scrimmage. He was aware that the hallucinations were not real and assumed that they were part of the task. Following the assessment, he discussed these hallucinations with his ophthalmologist and received a diagnosis of CBS (Table 1). 

**Table 1:** Charles Bonnet syndrome criteria Table created using the International Classification of Diseases 11th revision (ICD-11) for mortality and morbidity statistics criteria for visual release hallucinations (also called Charles Bonnet syndrome)

ICD-11: Visual release hallucinations (Charles Bonnet syndrome)	
Description	Exclusions
Complex visual hallucinations	Schizophrenia
In a person who has experienced partial or complete loss of vision	Other primary psychotic disorder
Exclusively visual	
Usually temporary	

Additional investigations included a referral to a neurovestibular clinic approximately 17 months post-mTBI. During this assessment, visual fields to confrontation in the right eye showed some monocular diplopia but no other significant abnormalities of the ocular and vestibular evoked myogenic potential. It was concluded that his vestibular symptoms were mainly provoked by visual triggers and indicative of postural perceptual persistent dizziness (PPPD). A magnetic resonance imaging of the brain and orbits prior to radiofrequency ablation therapy 23 months post-mTBI showed that optic nerves were symmetric in size with normal, bilateral signal intensity and an unremarkable optic chiasm. No changes were reported in comparison to a pre-injury CT head, which was also unremarkable.

The patient’s ophthalmic history included myopia and lattice atrophic holes in the retina, which had been treated with bilateral laser retinopexy, occasional left eye floaters, and color differences. Approximately 12 months after his bilateral laser retinopexy, the patient was referred to a retinal specialist due to ongoing double vision and floaters. No ophthalmic source for his complaints was found and no follow-up was indicated. He was advised that his symptoms may improve with time.

Since his mTBI, the patient has been assessed and treated for his PPCS and CBS symptoms by care providers in a variety of fields including ophthalmology, physical medicine and rehabilitation, physiotherapy (PT), occupational therapy (OT), speech and language therapy, and vestibular therapy.

Ophthalmology treatment initially included the use of binasal occlusion lenses, which helped with distance, and Fresnel prisms for blurred vision. However, the patient was still unable to focus his vision and remained symptomatic. He also tried orthoptist-led convergence exercises and vestibular therapy, but these approaches aggravated his symptoms. Wearing bifocals helped with his visual acuity and headaches. To treat the PPPD, his neuro-ophthalmologist referred him to clinical psychology as per treatment guidelines. 

For his PPCS, the patient attended physiatry appointments at a concussion clinic. There, he was re-referred to vestibular physiotherapy for convergence insufficiency and vestibulo-ocular reflex difficulties, a concussion peer support group, as well as PT and OT. As his PPCS symptoms have improved, he has been able to better engage in orthoptist-led convergence exercises.

The patient’s PPCS and visual hallucinations have persisted since the initial diagnosis. He reports missing people in crowds and noticing words moving when reading. When looking at blank backgrounds or at the sky with no clouds, he experiences complex hallucinations such as people walking behind a fence, a sheep staring at him, horses pulling up beside his car, and long hallways. For the most part, he can ignore the hallucinations. As a result, the frequency or duration of his hallucinations remain unclear as they are typically unnoticed. His most recent neuro-ophthalmology examination reported 20/30-2 visual acuity with correction and a small right eye cataract. The patient is ready to return to full-time employment, although he continues to struggle with periodic headaches, memory difficulties, and CBS.

## Discussion

This is a novel case of CBS in a patient with PPCS following mTBI and whiplash injury. Two previous case studies have reported CBS following TBI. The first patient reported hallucinations approximately one to two months post-TBI of unspecified severity. At 23 months post-injury, this patient experienced bradyphrenia, confusion, difficulty following directions, emotional withdrawal, tremors, cogwheeling, and dysdiadochokinesia. Following further imaging, they were diagnosed with dementia [12]. The second patient was admitted to the hospital one-month post-TBI with disorganized speech, inability to follow commands, aggression, nausea, poor appetite, and insomnia for three days. His Young Mania Rating Scale indicated mild mania. After stabilizing the patient’s acute symptoms, hallucinations subsided [13]. The differential diagnosis of CBS hallucinations included medication-related side effects and neurological or psychiatric disorders [17]; however, these criteria can be controversial [7]. Our case study adds to previous reports by providing further evidence that CBS may occur following brain injury. 

While not imperative for diagnosis, the incidence of CBS is higher in patients with worsening visual acuity or visual field deficits [18]. Our patient experienced a dynamic change in visual function following his mTBI, including exacerbated floaters, convergence insufficiency, and saccadic dysfunction prior to the onset of hallucinations. Although not previously associated with CBS, these PPCS are common in those who experience visual disturbances following mTBI [10,11,19].

Typically, CBS is managed by a multidisciplinary approach, which includes patient education, medical or surgical interventions where indicated, and vision rehabilitation [7]. A potential diagnosis of CBS was proposed and explained to our patient on the first complaint of hallucinations. Unfortunately, this is often not the case, with a recent study reporting that 55% of interviewed physicians were not aware of CBS, and 85% never discussed the possibility of hallucinations with their patients presenting with impaired vision [14]. This would suggest that there are a number of patients with CBS not receiving a diagnosis or appropriate education regarding their hallucinations, which is associated with poorer outcomes [15]. It is therefore important to increase awareness of CBS amongst physicians across all disciplines where visual disturbances may be reported to ensure timely assessment, diagnosis, and intervention. 

Following education, it is important to treat the underlying visual impairment [7]. Although research is limited on the effectiveness of orthoptist-led convergence exercises as a treatment for mTBI due to small sample sizes and poor study designs [20], there is some evidence that post-traumatic convergence insufficiency may improve with visual exercises. Santo et al. reported a significant improvement in near point of convergence following vision therapy in six of the seven studies reviewed [19]. Similarly, Gallaway et al. reported that 85% of the 41 participants with convergence insufficiency and 83% of the 18 participants with saccadic dysfunction who completed visual rehabilitation treatment had a successful outcome [11].

In addition to visual disturbances and hallucinations, our patient complained of headaches, fatigue, and reduced concentration. Although considered a potential treatment for CBS and oculomotor dysfunction following mTBI, orthoptist-led convergence exercises could not be tolerated by our patient until other symptoms were better managed. Following mTBI, symptom clusters vary amongst patients; therefore, a holistic evaluation and a personalized approach to management are recommended. 

## Conclusions

Over the last decade, there has been an increase in the instances of CBS in the literature, and a greater awareness of CBS is important moving forward. This case study further emphasizes the possibility of CBS developing following mTBI. In addition to that, we highlight the potential challenges in managing comorbid CBS and PPCS in an otherwise healthy adult male.
